# Stop Blaming me for What Others Did to you: New Alternative Masculinity’s Communicative Acts Against Blaming Discourses

**DOI:** 10.3389/fpsyg.2021.673900

**Published:** 2021-04-27

**Authors:** Tinka Schubert, Consol Aguilar, Kyung Hi Kim, Aitor Gómez

**Affiliations:** ^1^The Europe Center, Freeman Spogli Institute, Stanford University, Stanford, CA, United States; ^2^Department of Education, Universitat Jaume I, Castellón, Spain; ^3^Department of Education, Kyungman University, Changwon, South Korea; ^4^Department of Pedagogy, Faculty of Psychology and Educational Sciences, Universitat Rovira i Virgili, Tarragona, Spain

**Keywords:** communicative acts, communicative methodology, new alternative masculinities, feminist discourse, gender inequality, blaming

## Abstract

Some feminist discourses blame some men for gender inequality, gender domination, and gender-based violence. Some women use such discourse as a perfect scenario to criticize some men’s behavior. Indeed, they usually do so with Oppressed Traditional Masculinities (OTM) but not with Dominant Traditional Masculinities (DTM), who are the men who were violent with those women and with whom some of those women chose to have relationships. However, there have always been men who have been on the side of women and have never committed violence against them. Therefore, New Alternative Masculinities (NAM) reject being indicated as guilty of the violence committed against women by DTM. Through a communicative approach, applying six semi-structured interviews with a communicative orientation and a communicative data analysis of all information, this article explores both women’s communicative acts that blame OTM for what DTM have done to women and NAM’s reactions to these accusations to stop such blaming to make it possible to overcome hegemonic discourses.

## Introduction

Blaming discourses affect both women and men, but the literature has paid greater attention to the consequences of blaming women than men. Historically, blaming discourse has been used more against women in the feminist movement. An example of this type of discourse is the blaming of women for choosing to combine professional, personal, and family development because, as a consequence, they are not good mothers to their children (Jackson and Mannix, 2004; Bulbeck, 2010). In response to these blaming discourses, different scholars have characterized this type of discourse addressed to women as sexist and as promoting gender inequality (Bulbeck, 2010; Suarez and Gadalla, 2010).

In contrast, research has begun to consider that some discourses that derive from some feminist statements become blaming discourses toward men; these discourses assume that men are responsible for gender inequality and violence (Robinson, 2003; West and Zimmerman, 2009; Monteverde, 2014). Reactions to these blaming discourses against men continue to be scarce. Some of them have noted that this type of discourse of blaming men is developing into a prejudice toward men. Kiesling (2007) reviewed some feminist contributions and emphasized that some extreme statements communicated that the unique desire of men is to dominate women. In response to these extreme statements, some authors have indicated that not all women agree with this blaming discourse against men, and they have noted the need to distinguish between diverse types of feminist discourses because not all discourse is the same (Monteverde, 2014). For instance, international debates have emphasized the contributions of feminism to equality of differences and the promotion of solidarity between women from diverse cultural, academic, and age backgrounds to enhance social transformation through coherence with discourse and practice, and these contributions also defend egalitarian collaboration with men (Beck-Gernsheim et al., 2003; Joanpere and Morlà, 2019). This is in line also with Hooks (2000) notion of feminism understood as anti-sexism, “a male who has divested of male privilege, who has embraced feminist politics, is a worthy comrade in struggle, (…) whereas a female who remains wedded to sexist thinking and behavior infiltrating feminist movement is a dangerous threat” (Hooks, 2000, p.12).

However, at the same time, there is a profound need to analyze and identify how this discourse of blaming men has been used by some feminists to blame all men without distinguishing those who have perpetrated violence against women and those who have not. Considering this premise, the aim of this paper is to analyze how communicative acts (Soler and Flecha, 2010) performed by some women are reproducing this discourse of blaming men, especially those who have never engaged in gender violence, for instance, the blaming of Oppressed Traditional Masculinities (OTM hereinafter) for actions committed by Dominant Traditional Masculinities (DTM hereinafter). Additionally, this paper aims to make visible those communicative acts performed by New Alternative Masculinities (NAM, hereinafter), who are addressing these discourses to stop the blaming of them and to attempt breaking hegemonic discourses about them.

The article is divided in four sections. The first section provides a literature review that considers how the blaming discourse has affected women and men, and some similarities and differences that are found are discussed. The second section presents a description of the study performed and the data analysis applied. The third section presents the main findings related to those exclusionary communicative acts defined by those who are blaming men who have never committed violence toward women. Also, those transformative communicative acts that have led NAM to reject blaming discourses to overcome the hegemonic discourse that all men are guilty of gender inequality and violence, clarifying how DTM have committed violence, while they (non-violent men) never have. The fourth section provides a conclusion.

## State of the Art

### Blaming Discourse and Gender

There has been broad research on the influence of language on gender issues. In fact, gender is a social construction due to social interaction mediated by language (Bohan, 1992; Cameron, 2003; Eckert and McConnell-Ginet, 2003; Mills, 2006). In this sense, some scholars have noted the need to examine the uses of language that have a common sense or the types of uses that question beliefs that seem natural (Eckert and McConnell-Ginet, 2003). According to the studies reviewed, one of the research areas has been to evaluate the impact of language on gender interactions in the use of blaming discourses. This type of discourse has been addressed to women and men, both of whom have suffered prejudices and negative consequences due to blaming discourses (Robinson, 2003; Kiesling, 2007; Bulbeck, 2010; Thapar-Björkert and Morgan, 2010). The contributions reviewed have been classified in three main sections: first, definitions of blaming discourse are provided; second, blaming discourses addressed to women and men are analyzed; and finally, remarks considered in the study are provided.

To begin the examination of how blaming discourse has affected women and men, it is necessary to include a definition of what blaming discourse is. For this purpose, we have selected the definition of blaming discourse provided by Wodak (2006):

Blaming and denying constitute typical conversational patterns in conflict talk; such patterns are labeled “justification discourses.” The discourses of justification take place in public and private settings, in many written, oral, and visual genres. The explicitness of their linguistic realization depends on the formality of the conversation. Argumentation theory, speech act theory, and discourse analysis lend themselves best to analyzing justification discourses (Wodak, 2006, p. 59).

Considering Wodak’s (2006) definition, blaming discourse is typical in conflict talk, and it is used in public and private discussions and can be represented in different genres. Therefore, blaming discourse is a type of discourse used in conflict talk by those persons who are involved in the conversation. Within the category of private discussions, other scholars have identified how blaming is associated with the attribution of responsibility to others in the case of partner relationships: “An individual who acts in an abusive manner may ‘blame’ his or her actions on the behavior of the partner” (Scott and Straus, 2007, p. 853). Into the category of blaming discourse could be integrated the use of insults to blame the other. Wee (2015, p. 8) wrote, “insults are attempts by the speaker to get the target to feel worse about himself/herself (=the target) *via* implicating the speaker’s own superiority over the latter.” In fact, blaming others is an attempt to make the target feel worse, marking their inferiority relative to the speaker. Therefore, this evidence shows that blaming discourse intends to penalize the other. The characteristics of blaming discourses addressed to women and men found in the literature review are explained in the following sections.

### Blaming Discourse Addressed to Women

There are different types of blaming discourse addressed to women, but the literature selected has mainly focused on the type of discourse that blames women for being the victims of gender violence (Berns, 2001; Thapar-Björkert and Morgan, 2010) and, on the other side, attempts to combine professional, personal, and familiar roles in a way that is detrimental to the woman’s role as mother (Bulbeck, 2010). Attempting to determine who is using this blaming discourse addressed to women the most, the literature review included specifics that Socially Dominant Men are spokespersons for this type of discourse (Kelly et al., 2015).

Some scholars have analyzed how blaming is targeted mainly toward women in relationships in which gender violence occurs. Berns (2001) identified how patriarchal resistance defined domestic violence as a human issue in which women and men are responsible, but guilt is focused on women. In words of Berns (2001, p. 269), “Thus, although violence is degendered, blame is gendered.”. This contribution could be related to Thapar-Björkert and Morgan (2010), who, after reviewing some of the dominant discourse that sustains violence, conclude that the culture of blame disempowers women who are victims of domestic and sexual violence.

Other scholars who have identified that feminism has been blamed have noted that a discourse in which women who attempt to combine professional careers and motherhood cannot be good mothers (Bulbeck, 2010). In this sense, Jackson and Mannix (2004) collected the experiences of mothers who felt how this blaming discourse affected their daily life as mothers.

At this point, the question that remains is: Who is addressing this type of blaming discourse toward women? The blaming discourse is used mainly by men who are committing violence or who belong to dominant masculinities. For instance, Kelly et al. (2015) identified how Socially Dominant Men (men higher in Social Dominance Orientation, SDO) blame and despise women when they are rejected:

Men higher in SDO respond to romantic rejection with detrimental attitudes and behaviors, as a product of hostile sexism and belief that women ought to be disciplined for insubordination. Specifically, we argued that men higher in SDO would externalize the blame for rejection… (Kelly et al., 2015, p. 914).

This contribution coincides with the argument that violent men usually view their partners as malicious and blame them for relationship problems (Scott and Straus, 2007). Further, other scholars have identified by conversation analysis that some men sort men into two categories: those who hit women and those who do not (Stokoe, 2010). This type of masculinity corresponds to hegemonic masculinities that are learned in school, where deviance is characterized by different ranges of habits, including violence against other people (Hadjar et al., 2015).

The evidence found affirmed that there was no argument that justified this blaming discourse, there is no place for such a belief (Suarez and Gadalla, 2010). Therefore, blaming discourses toward women have been identified, such as sexist discourse that justifies gender violence (Bulbeck, 2010; Suarez and Gadalla, 2010).

### Blaming Discourse Addressed to Men

Compared with literature analyzing how blaming discourse has targeted women, literature about how men have been affected by blaming discourse is scarce. Kiesling (2007) reflected on this issue and wrote that some men are invisible, or when they are visible, they are always characterized by the dominant model. He remarked that some discourses contribute to men being presumed to be always coercive, and there is a need to examine how men are subjected to societal stereotypes as women are (Kiesling, 2007). It is important to highlight at this point how women are also under a process of submission to a coercive dominant discourse (e.g., through teen magazines, popular media, or TV among other things) that associates attraction with violence and influences socialization processes of women. NAM fight against this coercive dominant discourse (Puigvert et al., 2019).

Therefore, blaming discourse affects both genders, although there is much more literature on how this discourse has affected women than men. Blaming discourse categorizes women as responsible for being victims and men as perpetrators of the violence, in the literature reviewed. The aim of this paper is to analyze blame discourse addressed toward men and the reactions to it, given that there have been only scarce reflections on it.

Robinson (2003) performed a broad analysis of theories of masculinity and radical feminist theory to contrast the contributions and to determine the perspectives that could provide common ground for working together. Some of the contributions that she identified were related to some radical feminist contributions. These discourses blame men in their attempts to dominate women in gender, or they include other scholars’ contributions, based on the assumption of that all men are potential rapists; in words of Seidler (1994), “On the other hand, what about the radical feminist assumption that all men are potentially rapists?” (Seidler, 1994, p. 99). These sentences are typical of blaming discourse according to Wodak’s (2006) definition. Robinson (2003) argued that these assumptions do not help to construct a full dialog between some feminist scholars and masculinities authors, and there is a need to advance the dialog between both for advancing in gender issues.

These contributions collected by Robinson (2003) have also been shared by other authors. West and Zimmerman (2009) explained how this discourse is rooted in the contributions of the radical feminists of the early 1970s, when feminists from this perspective did not trust men who called themselves feminists; they saw them with great skepticism because they were men. In contrast, Monteverde (2014) stated that not all feminisms have this point of view and collected the contributions of feminists who were not blaming men, instead noting reciprocal responsibility in inequality.

Other scholars have indicated that masculinity analysis is contributing to the goal of equality and justice, but this contribution presents a difficulty because the majority of studies provide a negative definition of manhood, and it is necessary to construct an affirmative identity, but it is also necessary that scholars provide an analysis of how an affirmative identity of manhood can be achieved (Dowd, 2010). For instance, there have been contributions that have identified the social pressure on men to choose only between two categories: hard boys (tough) or soft boys (babyish, feminine; Georgakopoulou, 2005). However, there have been some contributions that have introduced NAM (Castro and Mara, 2014), providing a genuine alternative in which attractiveness and a clear statement against gender violence are combined in diverse masculine identities.

Our last observation is that we have identified a gap in the literature reviewed for this paper; we could not find any evidence for how the blaming discourse for the violence perpetrated by DTM is addressed by some women toward men who have never committed violence against women. There is evidence that some blaming discourses addressed toward men categorize all men as responsible for gender violence or gender inequality, while other scholars have rejected this blaming discourse as stereotype arguments, but none have analyzed in great depth how these blaming discourses are constructed and addressed toward men who have never committed violence. Another gap identified in the literature review was a profound analysis of how some men are rejecting this blaming discourse addressed toward them. For this reason, this paper contributes to closing this gap by analyzing how communicative acts (Soler and Flecha, 2010) performed by NAM (Flecha et al., 2013; Castro and Mara, 2014; Redondo-Sama, 2016; Joanpere and Morlà, 2019) are rejecting this blaming discourse, facing it and stopping this hegemonic discourse.

## Materials and Methods

### The Communicative Methodology Approach

This study was developed with communicative methodology (Díez-Palomar et al., 2014; Flecha and Soler, 2014; Vidu et al., 2014; Rios-Gonzalez et al., 2018; Redondo et al., 2020; Ruiz-Eugenio et al., 2020). The communicative perspective gathers contributions from several research traditions. Elements of phenomenology, constructivism, symbolic interactionism, ethnomethodology, dramaturgy, transcultural studies, dialogic action, communicative action, and dialogic learning contribute to this methodological approach. The communicative methodology is an answer to a society that increasingly demands egalitarian dialog and in which individuals increasingly adopt reflective and critical positions within our environment, providing the opportunity to contribute with their arguments to the development of the research process into an egalitarian position (Racionero and Padrós, 2010; Puigvert, 2012).

To respond to this dialogical turn of societies, the communicative methodology is based on an intersubjective epistemological conception, indicating that, from the point of view of reality (the phenomena studied in the research) it is the product of intersubjective agreements between individuals who use dialog to define the reality around them. In other words, the meanings given to objects around us are meanings that we share because we have discussed them and have reached agreements (Lopez de Aguileta, 2019). The interpretation of reality is based on these common agreements, in which researchers and subjects maintain their respective roles; researchers contribute to dialog with the academic background (the system), and the subjects contribute their knowledge, based on their experiences (the lifeworld; Habermas, 1984). In this sense, the construction of knowledge is always based on an egalitarian and intersubjective dialog between parts, linking theory and practice at the same time.

Taken from Habermas (1984) is the notion of social agents having the capacity of language and action and the dialogical creation of meaning through an egalitarian dialog. The most important aspect is the argumentation of the subjects and not the power relationships. The notion of common sense of social actors comes from Schütz and Luckmann (1973), and the notion of how subjects are not “cultural dopes,” which comes from Garfinkel (1967), is also crucial to Communicative Methodology. Thus, there is no interpretative hierarchy between researchers and subject; rather both are on the same epistemological level, the relationship, therefore, is not subject-object, but subject-subject (Beck et al., 1994; Gómez et al., 2006).

### Selection of Participants

The current research was fully approved by Community of Researchers on Excellence for All’s (CREA) Ethics Committee. Before being involved in the research, participants were contacted individually by the researchers, who fully informed them about the study. All of the interviewed people had in common that they have experienced the evolution of women and men working against what Connell conceptualized as hegemonic masculinity (Connell and Messerschmidt, 2005). They have been involved directly or indirectly in this fight, and they know perfectly well how women speak about men and how men react, both directly, if they have been involved in any association or movement against gender violence, and indirectly, if they have been close to militants in these movements, experiencing the fight with them. All the names used for the analysis of subjects’ communicative acts are pseudonyms.

The interviewed people had to meet at least one of the three following criteria:

- Be involved in movements against gender violence in the past, in the present or both;
- Have close relationships with representatives of the feminist movement; and
- Be involved in associations of NAM.

We present very briefly the profiles of the interviewed people:

#### Participant 1: Koldo

A university professor, he was involved in radical left-wing movements during the 70s, and he was very close to the feminist movement in the Basque Country, knowing many of their most important representatives. He experienced the emergence of the “difference feminism” and its coexistence with the “equality feminism.” He also knew the men fighting against gender inequality in different social movements in the Basque Country.

#### Participant 2: Carlos

A university professor, he experienced the influence of and the differences in feminism from the 90s until now. He has been very involved in the new masculinities’ movement in Madrid for more than 10 years, fighting against DTM within and outside the academy. He is professor of media and gender issues at the university.

#### Participant 3: Laura

A trainer, she was not directly involved in the feminist movement during the 80s, but she was involved in social movement struggles, and many friends of her were involved in the feminist movement in the Basque Country. She knows how the main feminist associations and the women assembled in the Basque Country work. She was not involved in this movement because she rejects the associations’ homogeneity and radicalization.

#### Participant 4: Nuria

She is involved in the social movement against gender violence. She was involved in the feminist movement in the 70s, and she is still participating. She has a valuable historical perspective on feminism and the current struggles. She was one of the founders of the Platform Against Gender Violence in Catalonia.

#### Participant 5: Antonio

A secondary school teacher, he has been involved for 5 years in men’s groups that follow the NAM approach. He has a direct vision of the oppression by some women of egalitarian men, and he now has many examples of how to overcome this situation of blaming men.

#### Participant 6: Pedro

A university professor, he has been involved in the movement against violence against women for more than 10 years, within men’s organizations. He is now an active member of a NAM association fighting against gender violence in Catalonia.

### Data Collection Techniques

Data were collected through semi-structured interviews (Alarcón et al., 2000) with a communicative orientation. The communicative approach allows researchers to interpret reality through egalitarian and intersubjective dialog with social actors, maintaining each of them in his or her respective role (Ramis et al., 2014; Melgar et al., 2020). In this case, the researchers contributed to dialog with academic backgrounds regarding communicative acts (Soler and Flecha, 2010) and NAM (Castro and Mara, 2014; Joanpere and Morlà, 2019), and contributions from participants were analyzed related to blaming discourse in this paper.

We conducted five face-to-face interviews, and one, for major reasons, was conducted online. We concreted the face-to-face interviews in natural scenarios for the interviewees. All of them were recorded and subsequently transcribed. The interviews lasted an average of 1 h. After an initial analysis of the speech acts, we met again with the men interviewed to go more deeply into some of the issues analyzed. Thanks to this second round, we concretely analyzed more in-depth the previous speech acts, and we provided more examples of blaming discourses against men.

### Communicative Data Analysis

The data were analyzed using communicative data analysis, which indicates the application of two dimensions: the exclusionary and transformative dimensions (Pulido et al., 2014; Redondo-Sama, 2016). The exclusionary dimension is defined by those barriers that prevent people from accessing concrete social benefits. In this paper, the exclusionary dimension is categorized through those communicative acts expressed by some women who blame those OTM who have not committed gender violence, based on acts committed by DTM. The transformative dimension consists of those components that help to overcome these barriers. In this paper, the transformative dimension is categorized trough communicative acts committed by NAMs that exemplified how they rejected being considered guilty of the violence committed against women by DTM.

## Results

### Exclusionary Communicative Acts: Blaming Men Who Never Have Committed Gender Violence

This section reports an analysis of those communicative acts addressed toward men who have never committed gender violence but who have been targets of blaming discourses for being a man. We selected examples of communicative acts in which blaming discourses were present in the interactions of women toward men in different times (70s, 80s, and currently) and in public (conferences debates) and private spaces (conversations between friends). This selection was based on evidence that the blaming discourse that began in the 70s is still present today, not only in some public feminist discourses but also in interpersonal relationships, as we can see in the following results. The analysis focused on verbal and non-verbal language, the social context of the interactions and the persons involved.

The first communicative act selected was provided by Koldo. He knows the feminist movement in the Basque Country since the 70s, and he experienced the change that occurred inside the feminist movement that brought it closer to difference feminism and moved it away from the prior equality approach. When starting to lead the movement, these women positioned themselves against all men, independent of whether these men were fighting in the feminist movement against oppression by DTM. Koldo remembered how some women involved in this feminism movement talked about men:

*The comments were like laughing at them because they are men (…) they had a concrete image of non-attractiveness, they were idiots to speak in a way (…) in general, they were looked down on; they were conceptualized as silly, and this fact was noted in all things -- in all human relations. In groups of both men and women, they occupied this position of silly men until the end (Koldo)*.

This communicative act exemplifies how the blaming discourse was addressed toward all men (“because they are men”) and how it included disrespect and insults (“they were idiots”). In Koldo’s words, these women did not consider that the men who were blamed by them were those who fought against gender inequality with them; these men also questioned DTM attitudes, but these women did not consider those men fighting in their feminist discourse.

In the 80s, the feminist discourse was very similar to that in the 70s. Laura was not directly involved in the feminist movement, but she had women friends who were involved in it. She remembers that they began to talk about who they were attracted to and why they were attracted to some men, and discourses about differences between dominant and egalitarian men were introduced in their conversations. Despite this discourse, Laura remembers that the dominant discourse that all men were equal remained: “The typical remark from one [woman] was that from all men whom I like I do not like anyone (…) how difficult it is to hook up; all men are bullies. This was the discourse” (Laura).

In this second communicative act, the blaming discourse contains a radical statement made by Laura’s friends (“all men are bullies”). This statement is a clear example of how blaming discourse creates prejudices and stereotypes that, in this case, harmed those men who had never committed violence.

However, these examples of communicative acts that blame men who never committed violence came not only from past decades. A more recent example was provided by Antonio. He is a member of an association based on the NAM approach that works against gender violence. During the interview, Antonio provided an example of a communicative act by a woman (Silvia) blaming a non-violent man (Salva) who is her friend and who always treats her well. This communicative act occurred during a conversation in a bar. Salva and Silvia were with other friends having conversations. Silvia maintained a long-term relationship with a boy with DTM attitudes, and she was involved with another DTM boy, and she used a blaming discourse toward Salva when he attempted to help her:

*In a concrete moment, Silvia said she had a headache, so Salva answered that she could take an aspirin. Her reaction, with disdain and a scornful face, was to shout at him: “That’s it, the advice! Women always want to be listened to by men, and men always give the same fucking advice!” (Antonio)*.

In the communicative act described by Antonio, the contemptuous use of language is very clear, together with the aggressive and violent attitude of the girl. In response to the advice offered in a friendly tone by Salva, one expression in the woman’s response is: “the fucking advice.” When we spoke to Antonio, he explained to us how Silvia’s DTM boyfriends (partner and former partner) did not listen to her at all, and she expressed her frustration to the boy who had never underestimated her (Salva). According to Antonio, she allowed the blaming of men, including Salva, who is not violent, and she addressed him with disdain, curse words, and an aggressive tone.

Another example along the same line was provided by Pedro. Due to his experience in men movements, Pedro has many examples of how some colleagues from the masculinities movement have experienced blaming discourses and how it has affected them. Pedro knows men’s groups working for equality in Catalonia, and he remembered how, in these groups, some men were analyzing themselves for contradictions that they probably had because of the patriarchy; many of them had suffered from blaming discourse for being men, even though they have never committed gender violence. The case provided by Pedro was about Miguel, a man who had never committed violence against his wife, but she (Alba) blamed him due to the specific feminist discourse she learned that all men are to blame for the patriarchy:

*Luis (a friend) told me that Miguel’s ex-wife said that he had committed violence (symbolic), and he felt that he had committed this violence (but there was not any evidence of it). He had a daughter, and Alba said that he had committed symbolic violence against his daughter. Alba used the discourse of some feminist women that they have learned very well. This discourse is based on blaming men -- all men and patriarchy; they (some feminists) put patriarchy in everything (Pedro)*.

Pedro said that Miguel felt so bad for being blamed, and even he believed that Alba had a reason. He interiorized the discourse of the women, and in the end, he believed that he really had committed violence, although he never did, according to Pedro. It is a very negative consequence of this process of blaming men with egalitarian values, harming those who never committed violence.

These communicative acts that exemplify blaming discourses also occur in public spaces, such as academic conferences. The influence of certain types of women who have propagated a specific type of feminism that present blaming discourses against men into academic discourse as well. Along these lines, Carlos, a university professor involved in the new masculinities movement, has participated in many academic events on gender issues over the last 10 years. During his interview, Carlos confirmed how most of the criticisms of men who fight against hegemonic masculinity came from women linked to some feminist movements who had elaborate discourses, as well as a violent attitude in the use of language and even gestures:

*I remember a situation when I was participating as a speaker at a conference on coeducation that one of the attendants, during the open debate, requested the floor. She was checking her notes; she was re-examining all of them one by one to note each of the words I said, speaking ironically and taking to extremes some of the examples that I used, creating caricatures, until giving her verdict: “a well-intentioned discourse” (Carlos)*.

Carlos was evaluated by the feminist point by point because she felt, as an expert in the area of coeducation, that Carlos had an egalitarian position. Carlos explained this situation as “the cotton test,” making a connection to the common situation when one is cleaning and passes cotton over the table to test whether it is really clean. He felt this way when the feminist spoke ironically against him:

*What attracted my attention the most was her discrediting tone, her position and even her posture: leaning back in the chair, with the pen in her hand, somewhat arrogant and overbearing (…) I was surprised by the anguish and unease she produced in me, maybe because it was unexpected (…). She waited to be the last participant, leaving no time to reply and generating an atmosphere of tension that could be fully perceived (Carlos)*.

As Carlos states, it is a language normally assigned to men for its aggressiveness but that began to be used by some feminists in the mid 70s and that is still used by some women today. In this example, the verbal language was offensive, as is the way in which the woman articulated her discourse (non-verbal language), denoting a type of power position in which the interlocutor (Carlos) was disregarded, and the spokesperson (feminist woman) was in a power position, which exemplified the typical argumentation of the blaming discourse:

*Nobody gave their opinion about her discourse, either to approve of it or to question it. I did not find solidarity. I wanted to, for instance, among other men attending the conference and working on gender issues. Worse is that even my answer was something submissive, I think, looking for her approval. Her ironic and disrespectful smile confused me. I wanted to disappear certainly. I never felt like that before (Carlos)*.

Then, as a consequence, the resulting atmosphere was tension in the space where it was produced and the generating of dissimilar reactions. Among men, as said by Carlos, there was neither support nor solidarity, and the interviewee himself was submissive in his answer, being at that moment an example of how OTM attitudes do allow for a reaction in the face of such an attack. The non-verbal language used by the woman, ironic and arrogant, together with her discourse, placed her above the others and caused Carlos not only to seek her approval but also to want to disappear from the event.

The blaming process against men who have never committed gender violence began during the 70s, with the process of change within the feminist movement. This blaming discourse has been maintained until today because there are some feminists and also non-feminist women who have attempted to blame egalitarian men for patriarchy and for the actions perpetrated by DTM. In the following section, we analyzed how men with NAM attitudes are now taking a position against this blaming discourse directed toward them.

### Transformative Communicative Acts: NAM Stop Blaming Discourses and Break Hegemonic Discourses About Men

This section reports an analysis of those communicative acts that respond to blaming discourses against men who never commit gender violence, overcoming the hegemonic discourse that all men are the same. We selected examples of communicative acts that occurred in private spaces (conversations between friends) and in public spaces (such as conferences and debates). This selection was due to evidence of how to stop this discourse of blaming men for perpetrating violence in both (personal and public spaces).

The first communicative act selected exemplifies that not all feminists share the same position as those feminists who are blaming men, as we analyzed in previous sections. In this sense, Nuria, a woman involved in the fight against gender violence in Catalonia, criticized the normalized discourse that many people currently engage in about how the situation of women is worse than in the past: “Any past time was better than now” (…). We are now in a good period (…) there is goodwill in the dialog between men and women. “Not all men believe in hegemonic masculinity” (Nuria).

Nuria, being a woman activist, made a clear statement (“Not all men are hegemonic masculinity”). This statement was the opposite of the feminist contributions analyzed in the previous section and confirmed that collaboration occurs between men and women (“There is goodwill of dialog between men and women”). This change in discourse could influence feminist debates and overcome the blaming discourses used by some feminists’.

However, the real change found in the fieldwork is the type of communicative act committed by some men who have acquired NAM attitudes to stop blaming discourses. In this sense, we recorded communicative acts provided by Antonio, Carlos, and Pedro.

Antonio provided us with an example of a communicative act in the face of blaming discourse. This type of discourse is sometimes applied in the manner in which women address men, seeming that they are angry with men. In this case, a woman interacted with a group of male friends (corresponding to a NAM model), and Sergio responded to her aggressive interaction toward them:

*The woman who was responsible for facilitating the proper footwear started to treat them in a less egalitarian manner. When forming the queue to ask for the footwear, she was asking them for their shoe sizes, but with an aggressive tone of voice and manners. Some of the boys chose their footwear, which was given to them in a very scornful way, without looking at their faces, until one of them (Sergio), when approaching the woman and before she had time to say anything, told her his shoe size, looking straight into her eyes and with a very secure tone of voice but without being aggressive. From that moment on, the woman set aside her aggressive tone of voice and moved on to more cordial treatment with them (Antonio)*.

Sergio stopped the woman’s aggressive manner of speaking with a secure attitude “but without being aggressive.” Facing these types of communicative acts is crucial, according to Antonio. This girl changed her attitude of being angry with the men because Sergio stopped it and did not allow her to continue.

These communicative acts that exemplify how to face blaming discourse are also present in academic debates occurring at conferences and workshops on gender issues. Pedro and Carlos talked about different academic events, conferences, and workshops in which men that favor gender equality received criticism from some women feminists. However, they also provided us with some examples of how some men who are acting according to the NAM model reacted with security and conviction in their answers, demonstrating the way to overcome the communicative acts that attempt to discredit them.

Pedro provided us with an example of a communicative act that occurred during a dinner with a speaker who was invited to a Conference of Masculinities organized by the City Council of Barcelona in 2005. A professor and principal of a school and an expert in sexuality (Daniel) were invited to this conference. He contributed to the debates and positions against the criticisms by some women of egalitarian man. After the conference, while having dinner, Daniel said to Pedro the following words that showed that he was so tired of the criticisms:

*Daniel said to me, “I’m fed up; I’m very tired. How could it be possible that, after 40 years, all men are guilty?” because recently, at this conference, some feminist authors were listed that continue with the discourse about patriarchy. I remember how Daniel said, ‘We are fed up. How can they blame you about patriarchy with twenty-odd that you have? What do you do in favor of patriarchy?’ His discourse was so simple but so clear*.

*Interviewer: But how did they say it? How did they express it?*

*In that way, you know, so simple. However, it is enough of that, blaming all men. They cannot keep blaming men for 40 years, it’s enough of this discourse. He was very outraged; he stayed in a good mood, but he was so tired (Pedro)*.

Daniel knows the feminist movement in the Basque Country, according to Pedro. He knows how they have been maintaining the same blaming discourse against egalitarian men from the 70s through today. This case occurred in Barcelona (Catalonia), but the argumentation and forms of the feminists were the same in both regions. These women reproduced the same discourse over time, and people like Daniel, who has been fighting against gender violence also since the 70s from an egalitarian position, is fed up; he is tired of it. The consequences can be detected in his verbal and non-verbal acts, because when he explained how it could be possible, he was quite outraged. As Pedro explained, his face and tone of voice denoted that he was very tired and outraged. This professor was clearly positioned against these feminist women during the conference, as well as afterward, when he was having dinner with our interviewee.

The last communicative act selected is an example provided by Carlos that exemplifies a reaction to blaming discourse used by a feminist woman in a public debate during a conference. In a Congress on Gender and Education in La Habana, just after a speaker from México, he was criticized by a feminist with a very aggressive and arrogant attitude. A well-known professor, who coordinated the Latin American Network of Masculinities, who was invited as the speaker and who was in the room at the same time, reacted as follows:

*He gave a very forceful answer. He established a discourse based on good arguments but also with determination, conviction, and a lot of security. What attracted my attention the most was his self-confidence in detailing the critical words said by the Argentinian woman who attempted to disqualify the Mexican speaker, accusing men of victimizing them and usurping the gender debate (…). After requesting the floor and with the microphone, he stood up, turned himself toward the audience to address the Argentinian woman, looking directly at her – who was getting smaller now, sitting among her colleagues -- and he rebutted the criticisms with respect but firmly and angrily to some extent. He was saying implicitly and explicitly, “I’m not going to allow you any criticisms formulated with the sole intention of disqualifying and denigrating from arrogance.” The way he expressed himself inspired great admiration, not only from me but also from many others (Carlos)*.

According to Carlos, he answered in such self-confident manner, with well-based arguments and without showing nervousness or needing to justify himself for being a men (“I’m not going to allow you any criticisms formulated with the sole intention of disqualifying and denigrating from arrogance”), the Argentinian woman had no way to counteract his arguments but, in contrast, was ashamed (“who was getting smaller now, sitting among her colleagues”). The non-verbal language of the man was crucial in this sense because, by the mere fact of standing, turning, and looking at her firmly when rebutting her, he generated the sought effect on her. He was also reinforced because he generated on the floor a sense of admiration for him.

Therefore, the communicative acts that respond to blaming discourses obtain the result of breaking the hegemonic discourse that all men are the same. NAM’s reactions put things in order, and they stop this discourse by not allowing men to be treated in disrespectful manner for things done by DTM.

## Discussion

This paper has contributed to advancing the knowledge in two aspects: first, that there exists a blaming discourse directed at men in general, blaming all men, those who perpetrate violence and those who fight against it for violence committed against women; and second, that there are some men, that can be classified as NAM whose reactions are stopping these blaming discourses based on action committed by DTM. In this sense, the analysis of communicative acts (Soler and Flecha, 2010; Rodríguez-Navarro et al., 2014; Carrillo et al., 2017; Rios-Gonzalez et al., 2018) is crucial for identifying the types of communicative acts that are either exclusionary and that promote blaming discourses (Wodak, 2006) against men who have never committed gender violence or transformative in the sense that they counter the blaming discourse, thus, helping to draw a line between who to blame and for what.

The results show how some discourses rooted in the radical feminists movements of the 70s (which are still present today) have promoted those blaming discourses regarding gender inequality toward all men (Robinson, 2003; West and Zimmerman, 2009), without distinguishing those who have contributed to it from those who have not. The scientific literature has evidenced that some men even part from feminist movements to continue their struggle against violent men on their own as they can no longer account to these women’s request. From being allies in the feminist movement they have been pointed at as enemies for the single fact of being men. However, the literature has also clearly identified how men who used blaming discourses against women also belonged to a dominant model and committed violence (Scott and Straus, 2007; Kelly et al., 2015).

The present research contributes to the gap on how some men clearly position themselves as allies to the feminist movements and how for this specific reason they are subject to a blaming discourse perpetrated by women, especially feminist women following a certain discourse to discredit them. Yet, more often than not, these women would not dare using the same discourse with men who perpetrate violence.

Men who represent the DTM are the perpetrators of gender-based violence and not the ones representing OTM. Solnit (2014) argues that violence is not based on race, nationality, or religion, but on gender. She highlights how men are the ones who exercise violence, but not all, most of them are not violent and they are also suffering from violence exercised by others. The positioning of men who represent the NAM against other men who exercise this violence and against some women who exercise blaming discourses against men is fundamental in the struggle to overcome gender violence (Redondo-Sama, 2016).

In contrast, in the results from the fieldwork, there were only examples of how the selected communicative acts performed by women from some feminist movements blamed men who have never committed gender violence. On the other side, men who take a stand and do not allow themselves to be treated in a contemptuous or blaming manner perform transformative communicative acts to stop the blaming discourse from being addressed toward them, and in this way, they break the hegemonic discourse about men.

Finally, we want to highlight future research lines. It would be more insightful to have a greater number of participants to carry out fieldwork. In that sense, it would be interesting to think about the possibility of combining different collective and individual data collection techniques. In this way, it would be possible to analyze blaming discourses against men by triangulating individual and group information.

Blaming discourses affect younger people. For this reason, it would be interesting to work with young people in future research. In the same way, it is important to develop fieldwork in other regions. If we worked with this target group, it would be possible to fight against blaming discourses at an early stage. Future research could also incorporate social impact as one of the main research variables. If we looked for a positive social impact on young people from the very beginning of our research, it could be easy to fight against these blaming discourses.

### Limitations

The information extracted and analyzed from the semi-structured interviews showed some situations and actions of other people, because they have not been experienced firsthand by the interviewed person. This fact affected the narrative and the concretion of the communicative acts. To overcome this limitation, we contacted some of the interviewees a second time with a selection of possible communicative acts, asking for more concrete details and also sharing the first interpretation of the information. A second limitation lays in the regional context of the interviewees. We conducted concrete interviews of people who live in the Basque Country, Catalonia, and Madrid, and the information provided was mainly centered on these regions. The analysis of the feminist movement and also of men’s movements against gender inequality was located in these areas, and at no time are they extended to all women of the various feminist positions, nor of other places.

## Data Availability Statement

The datasets generated for this study are available on request to the corresponding author.

## Ethics Statement

The studies involving human participants were reviewed and approved by The Ethics Committee of the Community of Researchers on Excellence for All (CREA), University of Barcelona. Written informed consent for participation was not required for this study in accordance with the national legislation and the institutional requirements.

## Author Contributions

TS, KK, CA, and AG made substantial contributions to the conception of the manuscript, searching the literature, drafting the article, and revising it critically. AG was responsible for data collection and contributed to the discussion and conclusions. TS conceptualized and designed the article and revised and approved the manuscript. KK and CA collaborated in data analysis and in elaborating the article. All authors contributed to the article and approved the submitted version.

### Conflict of Interest

The authors declare that the research was conducted in the absence of any commercial or financial relationships that could be construed as a potential conflict of interest.
